# Experimental measurement and modeling of asphaltene adsorption onto iron oxide and lime nanoparticles in the presence and absence of water

**DOI:** 10.1038/s41598-022-27335-z

**Published:** 2023-01-04

**Authors:** Sajjad Ansari, Mohammad-Reza Mohammadi, Hamid Bahmaninia, Abdolhossein Hemmati-Sarapardeh, Mahin Schaffie, Saeid Norouzi-Apourvari, Mohammad Ranjbar

**Affiliations:** 1 Department of Petroleum Engineering, Shahid Bahonar University of Kerman, Kerman, Iran; 2 State Key Laboratory of Petroleum Resources and Prospecting, China University of Petroleum (Beijing), Beijing, 102249 China; 3 Department of Mining Engineering, Shahid Bahonar University of Kerman, Kerman, Iran

**Keywords:** Energy science and technology, Engineering, Chemical engineering

## Abstract

Asphaltene precipitation and its adsorption on different surfaces are challenging topics in the upstream and downstream of the oil industries and the environment. In this research, the phenomenon of asphaltenes adsorption in the presence and absence of water on the surface of magnetite, hematite, calcite, and dolomite nanoparticles (NPs) was investigated. Five asphaltenes of different origins, four NPs as adsorbents and Persian Gulf water were used for three-phase (asphaltene/toluene solution + NPs + water) experiments. Characterization of asphaltenes and NPs was performed using Fourier transform infrared spectroscopic (FTIR), dynamic light scattering (DLS), elemental analysis, and field emission scanning electron microscopy (FESEM). Adsorption experiments were performed in two- (asphaltene/toluene solution + NPs) and three-phase systems. The results showed that the most effective parameters for asphaltene adsorption onto these NPs are the asphaltene composition, namely nitrogen content, and the aromaticity of asphaltenes. The significant effects of these parameters were also confirmed by the relevancy factor function as a sensitivity analysis. In the competition of asphaltene adsorption capacity by NPs, iron oxide NPs had the highest adsorption (Magnetite NPs > Hematite NPs > Calcite NPs > Dolomite NPs). From the results of the experiments in the presence of water phase, it could be pointed out that the asphaltenes adsorption onto the NPs was accompanied by a decrease compared to the experiments in the absence of water. The modeling also showed that physical adsorption has a significant contribution to the asphaltenes adsorption on the surface of iron oxides and lime NPs. The results of this research can assist in a better understanding of the asphaltene adsorption phenomenon and the role of iron oxide and lime NPs in solving this problem.

## Introduction

Asphaltene is the heaviest component of crude oil, which was identified by a French researcher Boussingault^1^. Asphaltenes are soluble in aromatic solvents, but they are not soluble in paraffinic solvents with a low boiling point, such as n-heptane and n-pentane^2,3^. Asphaltenes have a multi-ring cluster structure that is alternately replaced by alkyl groups and has heteroatoms (nitrogen, sulfur, and oxygen) and amounts of metal elements (nickel, vanadium, etc.)^4,5^. Asphaltene, as the most aromatic and polar colloidal component of crude oil, can cause various problems in the upstream and downstream of oil industries and the environment^6^. Asphaltenes are stabilized in colloidal state by resins in crude oil^2,7^. When these resins in crude oil are removed due to factors such as pressure, temperature, and changing the composition of crude oil, asphaltenes can flocculate and create large enough particles. Then, the polarity of asphaltene and various functional groups cause it to be adsorbed on different solid surfaces and create damage to the formation (change in wettability, plugging of pores in the reservoir rock, followed by a decrease in permeability), well column and wellhead equipment (oil lines), which can lead to serious economic costs^8–10^. From an environmental point of view, the adsorption of asphaltene molecule on the soil in places where crude oil is spilled on the surface of the earth, can cause destructive environmental effects and according to the type of adsorption (physical or chemical adsorption) eliminating this phenomenon is a difficult task^6^. This phenomenon is so important that many industry and university researchers have investigated it to provide suggestions to reduce its problems and even make optimal use of its potential. In recent years, due to the great success of Nanoparticles (NPs) in engineering sciences, the use of NPs to solve asphaltene adsorption has been welcomed by researchers. NPs have had good success in solving this problem due to their functionalization ability, very high surface-to-volume ratio, high surface adsorption ability, etc.^11–13^.

Dudášová et al.^14^ investigated the effect of different NPs on the adsorption of asphaltenes. The results show that the amount of adsorption is highly dependent on the type of adsorbent (type of NPs). Nassar et al.^15^ used iron oxide NPs, namely, magnetite, cobalt oxide, and nickel oxide to adsorb asphaltene and remove it from heavy oil. From their results, it was found that used NPs have a high adsorption capacity, and their capacity were classified as follows: NiO > Co_3_O_4_ > Fe_3_O_4_. In another study by Nassar et al.^11^, they investigated the adsorption of asphaltene onto metal oxide NPs (Fe_3_O_4_, Co_3_O_4_, MgO, NiO, CaO, and TiO_2_). The results revealed that the adsorption capacity depends on the molecular weight of asphaltenes. Madhi et al.^16^ surveyed the asphaltene adsorption onto three NPs (SiO_2_, MgO, and Al_2_O_3_). Based on their results, physical adsorption plays a critical role in the surface adsorption mechanism of asphaltene onto NPs. Ansari et al.^6^ compared the amount of asphaltenes adsorption on the surface of dolomite NPs and microparticles and showed that dolomite NPs have a higher adsorption capacity than dolomite microparticles, and this higher adsorption capacity is because of the greater specific surface area of NPs compared to microparticles. Bahmaninia et al.^17^ investigated the impacts of hydrophobic and hydrophilic quartz NPs on asphaltene adsorption in the existence and absence of water. The results showed that overall, quartz NPs adsorb less asphaltene in the presence of water than in the absence of water. Also, the result of Gonzalez and Taylor's^18^ research showed that the presence of water in asphaltene uptake process decreases the amount of adsorption. In addition, the studies of other researchers on the adsorption of asphaltenes on the surfaces of NPs (Fe_3_O_4_/TiO_2_, Fe_3_O_4_/Chitosan, Fe_3_O_4_/SiO_2,_ γ-Fe_2_O_3_, α-Fe_2_O_3_, NiO/AlPO-5, NiO/ZSM-5, Fe_2_O_3_, Fe_3_O_4_, and Al_2_O_3_) all showed their high adsorption capacity^19–22^.

**Figure 1:**
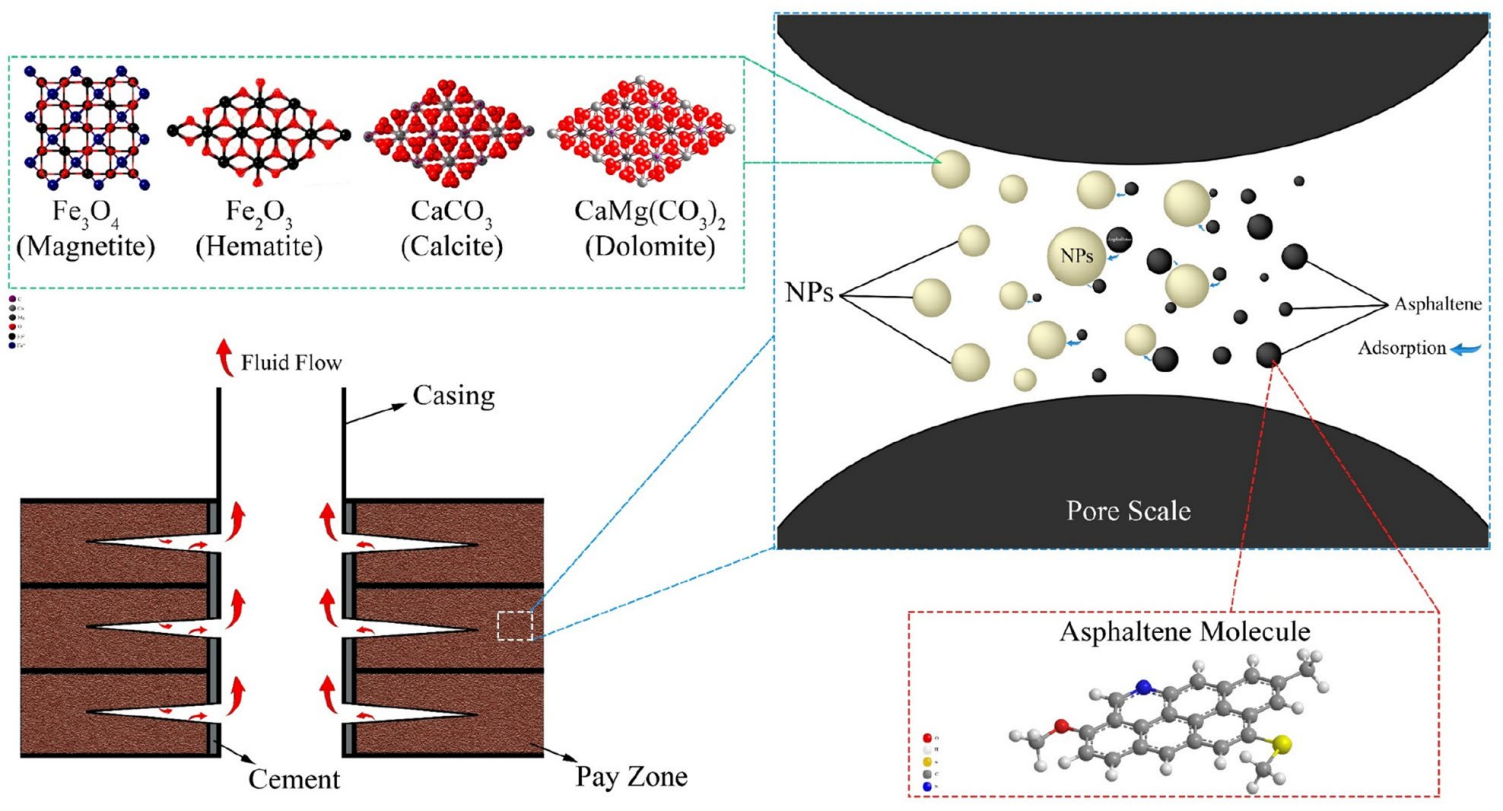
Molecular illustration of the issue under investigation in the current work.

According to many types of research that have been carried out in the field of asphaltene adsorption on various adsorbents including NPs, the effect of many parameters has been identified, which has been investigated in detail in the review works of Adams^23^, Mazloom et al.^24^, and Tazikeh et al.^25^. Based on numerous investigations regarding the phenomenon of asphaltene adsorption, the need for a comprehensive and comparable study between different NPs with asphaltenes of different origins is sensed. Considering that adsorption is a competitive phenomenon and strongly depends on the composition of adsorbate materials as well as the competition of different adsorbate units in the adsorption process, it seems that there are still many ambiguous issues about the composition of diverse asphaltenes and their competition with other molecules such as water molecules for adsorption on various NPs that require more laboratory investigations. A comparative study of several NPs with asphaltenes of different origins can help to better understand this complex phenomenon and clarify various unanswered questions of this phenomenon. Also, due to the coexistence of water and oil in real conditions in the reservoir, the effect of water on asphaltene adsorption has always been important and should be investigated in details. In this work, we tried to fill the mentioned gaps in this realm. It should be noted that this work is a continuation of our systematic studies on the adsorption of various asphaltenes onto different surfaces: dolomite microparticles and NPs^6^, hydrophilic and hydrophobic quartz microparticles and NPs^17^, and magnetite microparticles^26^. The molecular sketch of the problem under investigation in the current research is illustrated in Fig. 1. This figure shows that the asphaltenes in oil with functional groups are absorbed by NPs, which can reduce asphaltene precipitation and deposition in the porous medium. Although here only a schematic of the problem considered in this research is depicted and the aim is not to investigate the molecular structure of asphaltene; for this purpose, interested readers could refer to the literature^27–29^.

In this study, considering the good success of iron oxide NPs in asphaltene adsorption, we used magnetite (Fe_3_O_4_) and hematite (Fe_2_O_3_) NPs compared to two important lime NPs, i.e., dolomite (CaMg(CO_3_)_2_) and calcite (CaCO_3_) NPs. For better understanding of adsorption phenomenon, five asphaltenes of different origins are used. Fourier transform infrared spectroscopy (FTIR), dynamic light scattering (DLS), field emission scanning electron microscopy (FESEM), and Elemental analysis are employed to characterize asphaltenes and NPs. Moreover, Persian Gulf water is utilized for three-phase tests. A sensitivity analysis is performed using the relevancy factor to investigate the effect of various asphaltene parameters on their adsorption onto the NPs. Finally, four adsorption isotherm models namely, Langmuir, Freundlich, Dubinin-Radushkevich, and Temkin are applied for modeling the adsorption results.

## Materials and methods

### Materials

Five types of asphaltenes were extracted from five diverse types of oil from Iranian fields using the ASTM (D-2007-80) method^30^. Persian Gulf water with pH 7.9 containing Cl^−^, SO_4_^2−^, HCO_3_^2−^, K^+^, Na^+^, Ca^2+^, and Mg^2+^ ions and total dissolved solids of 41 g/l^31^ was utilized to perform three-phase (asphaltene/toluene solution + NPs + water) experiments. Hematite NPs (99.5% pure) with a mean particle size of 20 nm (based on brochure information) and calcite NPs (99.5% pure) with a mean particle size of 60 nm (based on brochure information) were purchased, and dolomite and magnetite NPs were produced from their source rocks using a mechanical mill (Fritsch, PULVERISETTE, Germany) in the laboratory. The method of producing dolomite NPs has been presented in detail in our recent work^6^. The size of nano dolomite particles by DLS analysis is in the range of 200–530 nm, and by SEM analysis, the particle size is 60 nm. Moreover, mechanical mill was used to produce magnetite NPs. First, the magnetite stone was crushed into fine iron powder (30–70 μm). Wet iron powder along with steel balls with a mass ratio of ball to powder 50:1 was poured into the cups of the machine and milled at a speed of 300 rpm for 24 h. After that, the milled product is dried under vacuum. In this method, the mass reduction of steel balls during milling is in such a way that it accounts for almost half of the total weight of iron in the system. The presence of distilled water and iron pellets plays an essential role during milling and make Fe_3_O_4_ form^32^. The average size of NPs was found to be 91 nm by DLS analysis. It should be noted that the DLS analyzer calculates the hydrodynamic diameter of the particles, which is a significant amount at the level of NPs.

Examining the effect of adsorbent particle size on asphaltene adsorption is a parameter that has been mentioned in many studies, we have covered the effect of this parameter completely in our previous paper from micro to nano ranges^6,17^. The average particle size of hematite, calcite, dolomite, and magnetite NPs obtained by SEM was about 40, 55, 60, and 50 nm, respectively. This indicates that the range of NPs used was almost similar and their small difference would not have a considerable effect on the adsorption process. As mentioned, the main purpose of this article is to address the impact of asphaltene composition and also to create a competitive environment between asphaltene and water for adsorption on NPs regardless of their size.

### Methods

#### Characterization of asphaltenes and NPs

FTIR analysis of asphaltenes was done and described comprehensively in our previous works^6,26^ and here it is described briefly. FTIR analysis (Brucker, TENSOR 27, Germany) was used to identify the structures and functional groups of asphaltenes. In this analysis, asphaltene samples were mixed with potassium bromide powder. Also, samples were analyzed in the range of 400 to 4000 cm^−1^ with 32 scans and a resolution of 4 cm^−1^. Elemental analysis (Elementar, Vario MACRO, Germany) was used to determine nitrogen, carbon, sulfur, and hydrogen elements in asphaltenes samples. The oxygen content and H/C ratio of samples was calculated from CHNS values. DLS analysis (Malvern, ZEN3600, England) was applied to check the particle size of asphaltene samples and NPs. The samples were prepared in the solvent for 5 min at 25 °C. For asphaltene samples, toluene solvent and non-ionized water NPs were used. FESEM analysis (HITACHI, S4160, Japan) was used to identify the surface characteristics and morphology of NPs.

#### Two-phase adsorption experiments

Adsorption experiments were carried out by adding 0.1 g of NPs to 10 ml of different concentrations of asphaltene/toluene (0, 100, 200, 500, and 1000 ppm). The samples were placed in an incubation shaker for 18 h at a speed of 250 rpm at room temperature and atmospheric pressure^6^. The adsorption of asphaltenes on NPs was measured by UV–visible spectrophotometer. It should be noted that the adsorption measurement was performed at the wavelength of λ = 410 nm, which renders sufficient sensitivity for measurement due to the presence of metal porphyrins in asphalts^22,33,34^. Finally, the amount of asphaltenes adsorbed on NPs surface was calculated by Eq. (1). In this equation, *q* is the asphaltene uptake (mg/g), *V* shows the volume of solution (L), *C*_*0*_ is the initial concentration of asphaltene solution (mg/L), *C*_*e*_ shows the equilibrium concentration (mg/L), and *m* denotes adsorbent mass (g)^6^.

1 $$q=\frac{\left({C}_{0}-{C}_{e}\right)V}{m}$$

#### Three-phase adsorption experiments

Three-phase tests were performed similarly to two-phase tests, with the difference that water was added to the system in three-phase tests. In these experiments, considering a system having water and asphaltene/toluene solution in a ratio of 1:1, NPs were added to it for investigating the competitive adsorption performance of asphaltenes and water. Here, two and three-phase systems contain an equal mass of asphaltenes in the whole solutions, and therefore, the effect of the water phase on the adsorption of asphaltenes by NPs was studied. It is noteworthy that utilizing a shaker-incubator, the mixture of asphaltene/toluene solution and water is stirred before the addition of NPs and after their addition during the continuous experiment^17,26^.

#### Adsorption isotherm models

Dubinin-Radushkevich, Temkin, Freundlich, and Langmuir models were used for modeling the adsorption processes of asphaltenes on NPs. The nonlinear and linear forms of the mentioned models were shown in Eqs. (2) to (5), respectively. For more information about the adsorption models used in this study, you can refer to the literature^35–37^.

Langmuir:

2 $$\mathrm{Nonlinear\, form:} {q}_{e}={q}_{m}\frac{{k}_{l}{C}_{e}}{{1+k}_{L}{C}_{e}} \quad \mathrm{ Linear\, form:} \frac{{C}_{e}}{{q}_{e}}=\frac{1}{{K}_{L}{q}_{m}}+\frac{{C}_{e}}{{q}_{m}}$$

Dubinin–Radushkevich:

3 $${\mathrm{Nonlinear \, form:}\,\, q}_{e}=\left({q}_{s}\right)exp\left({-k}_{ads}{\varepsilon }^{2}\right) \quad \mathrm{ Linear\, form:} \,\, ln\left({q}_{e}\right)=ln\left({q}_{s}\right)-{k}_{ads}{\varepsilon }^{2}$$

Freundlich:

4 $${\mathrm{Nonlinear\, form:} \,\, q}_{e}= {k}_{F}{C}_{e}^\frac{1}{n} \quad \mathrm{ Linear\, form:} \,\, {\mathrm{log}(\mathrm{q}}_{\rm{e}})=\mathrm{ log}\left({\mathrm{k}}_{\rm{F}}\right)+\left(\frac{1}{\mathrm{n}}\right){\mathrm{logC}}_{\rm{e}}$$

Temkin:

5 $$\mathrm{Nonlinear\, form:}\,\, {q}_{e}=\frac{RT}{{b}_{T}}ln\left({k}_{T}{C}_{e}\right) \quad {\mathrm{ Linear\, form:}\,\, q}_{e}=\left(\frac{RT}{{b}_{T}}\right)ln\left({k}_{T}\right)+\left(\frac{RT}{{b}_{T}}\right)ln\left({C}_{e}\right)$$

## Results and discussion

### Characterization of NPs

**Figure 2:**
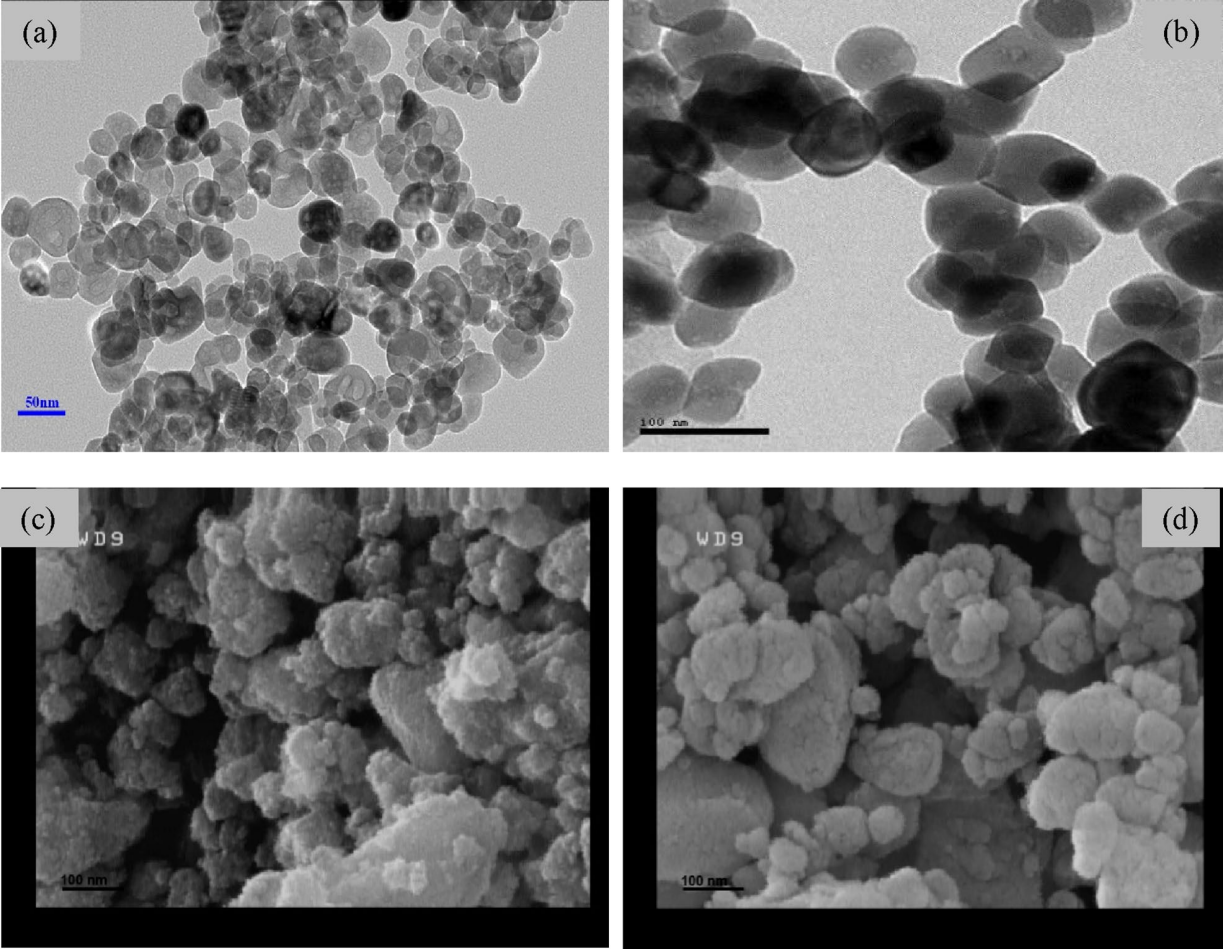
FESEM analysis of (**a**) hematite^38^, (**b**) calcite^39^, (**c**) dolomite, and (**d**) magnetite NPs.

Hematite NPs are red in color, calcite NPs are white, dolomite NPs are yellowish brown, and magnetite NPs are black. Figure 2 shows the FESEM analysis images of NPs. According to Fig. 2, hematite NPs have an almost spherical shape and calcite NPs have a cubic shape. Due to the fact that dolomite and magnetite NPs are prepared by a physical method, i.e., using a mechanical mill, they have caused irregular shapes in the shape of the particles. It is worth mentioning that dolomite NPs were produced in our previous work and used for two-phase static adsorption experiments, and the method of producing dolomite NPs has been presented in detail in our previous study^6^.

### Characterization of asphaltene

The complete characterization of asphaltenes has been fully reported in our previous works^6,17,26^ and a brief description is provided here again. Elemental analysis was done to check the content of five asphaltenes, and the results are presented in Table 1. According to Table 1, As-1 has more heteroatom content (NSO) than other asphaltenes. The content of heteroatoms (total NSO) indicates the polarity of the asphaltene, and the higher this value in the asphaltene, the higher the polarity of the asphaltene^26,40^. Therefore, As-1 owns the highest polarity and As-3 owns the lowest polarity among the five asphaltenes. The H/C ratio indicates the aromatic nature of asphaltene, the lower its value, the more aromatic the asphaltene is^41,42^. According to Table 1, As-2 has the most aromatic nature (aromatic rings) and As-4 has the least aromatic nature compared to other asphaltenes.

**Table 1:** The elemental analysis of five asphaltenes^17,26^.

Samples	N (%)	S (%)	O (%)	NSO (%)	H (%)	C (%)	H/C (molar ratio)
As-1	1.26	5	4.48	10.74	8.64	80.62	1.276
As-2	1.47	3.07	4.53	9.07	8.35	82.58	1.204
As-3	0.94	1.84	4.22	7	9.03	83.97	1.281
As-4	0.82	2.66	4.98	8.46	9.19	82.35	1.329
As-5	0.97	3.02	5.36	9.35	8.79	81.86	1.279

**Figure 3:**
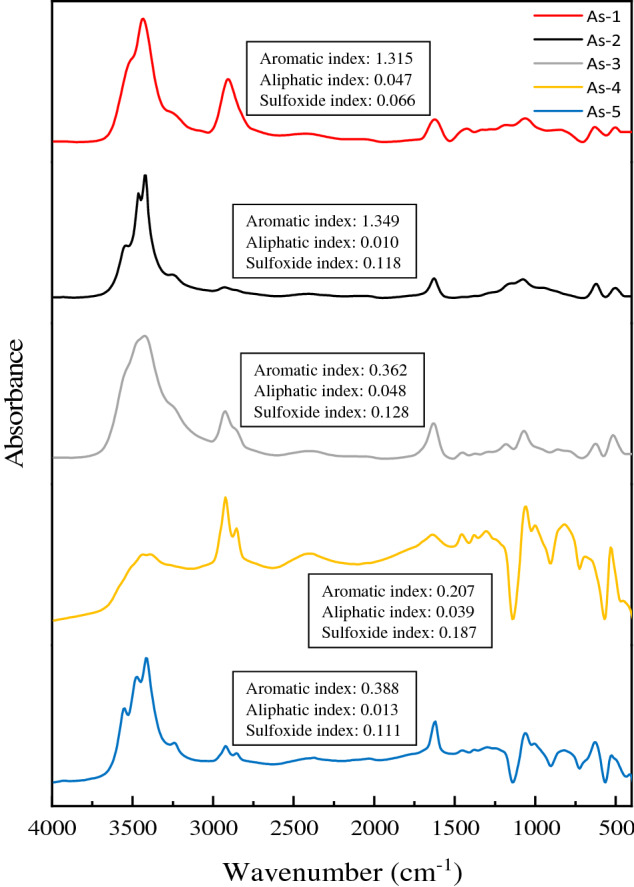
FTIR spectrum of asphaltene samples^26^ and structural parameters of asphaltene samples based on FTIR analysis^17,26^.

As stated earlier, FTIR analysis of asphaltenes was described comprehensively in our previous works^6,17,26^ and here it is described briefly to make it sensible for readers. In order to investigate the functional group of asphaltene, which is of great importance, FTIR analysis was done from the five asphaltenes studied. Figure 3 shows the spectra of this analysis for five asphaltenes. For better comparison of FTIR results, it is recommended to normalize all spectra. The normalization process was according to the asymmetric stretching of the C–H bond in the CH_2_ (methylene) groups at the wavelength 2923 (cm^−1^), which was seen in the five samples of this peak. Table S1 reports all the significant peaks identified and all the functional groups of the five asphaltenes. In addition to identifying the functional groups of asphaltenes, the structural properties of asphaltene samples can be identified by calculating some indices, which are defined in accordance with the area under the peaks of the FTIR analysis spectrum. It can provide useful insight in investigating the adsorption behavior of asphaltenes. Aromatic index = $\frac{{A}_{1600}}{{A}_{814}+{A}_{743}+{A}_{724}}$, aliphatic index = $\frac{{A}_{1460}+{A}_{1376}}{\sum A}$, and sulfoxide index=$\frac{{A}_{1030}}{\sum A}$ were calculated in this study^43,44^. In the above indices, the letter *A* indicates the area under the peak at wavelength x. Where the subscript and *A* numbers show the wave number corresponding to the peak and the peak region in the adsorption spectrum, respectively^43,44^.

6 $$\sum A={A}_{724}+{A}_{743}+{A}_{814}+{A}_{864}+{A}_{1030}+{A}_{1376}+{A}_{1460}+{A}_{1600}+{A}_{1700}+{A}_{2862}+{A}_{2923}+{A}_{2953}$$

The results of indices calculations are presented in Fig. 3. According to Fig. 3, As-2 then As-1 has the highest aromatic value and the lowest aromatic value is related to As-4, which is consistent with elemental analysis (Table 1). Among the five asphaltenes, As-2 has the least aliphatic compounds and As-3 has the most aliphatic compounds. Also, there is the highest frequency of S=O bonds in As-5, while the least of these compounds is in As-1.

**Figure 4:**
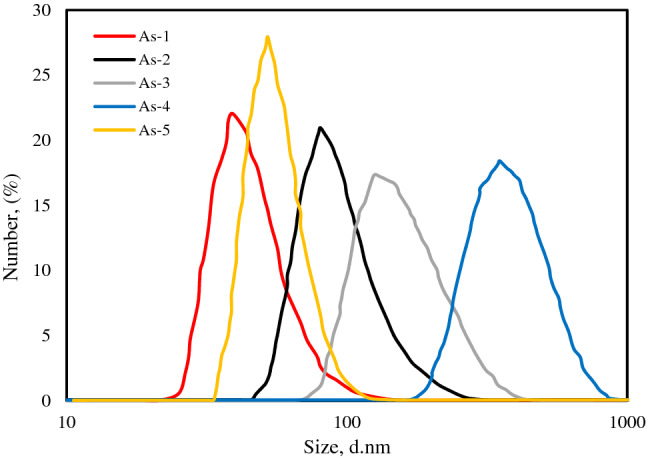
Particle size distribution of five asphaltene samples^26^.

DLS analysis was carried out in our previous works^6,17,26^, and here just a brief explanation of that is provided in order to use in the analysis of the current work. Figure 4 depicts the particle size distribution of five used asphaltene samples in this work obtained by DLS analysis. According to these results, the mean particle size of asphaltenes was calculated as 46 ± 11, 96 ± 25, 161 ± 44, 380 ± 92, and 55 ± 10 respectively. Based on the results, As-1 has the smallest and As-4 owns the largest particle size in the comparison with other samples. It is noteworthy that a relationship among the asphaltenes average particle size and the sulfoxide index of FTIR analysis, such that the greater the asphaltene sulfoxide index, the greater the average particle size. Sulfoxide considered a very polar group, thus, the higher frequency of it in a sample, the lower the asphaltene solubility in toluene and the larger the asphaltene particle size in DLS analysis^29^.

### Two-phase asphaltene adsorption experiments

**Figure 5:**
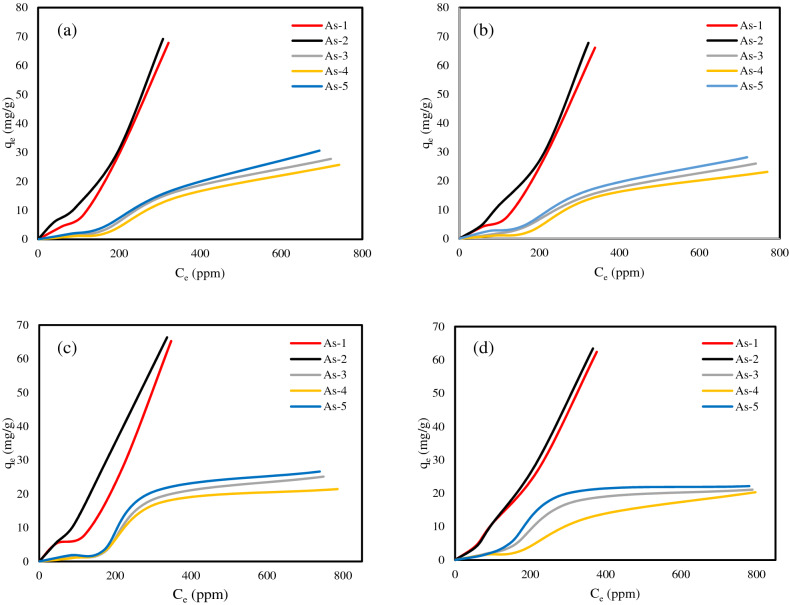
Isotherms of asphaltenes adsorption onto (**a**) magnetite NPs, (**b**) hematite NPs, (**c**) calcite NPs, and (**d**) dolomite NPs.

The results of the two-phase adsorption experiments are presented in Fig. 5. According to Fig. 5, As-2 has the most, and As-4 has the least tendency to be adsorbed on the surface of all NPs. According to the tendency of asphaltenes to be adsorbed on the surface of all NPs, they are ranked as follows: As-2 > As-1 > As-5 > As-3 > As-4. These results show that the adsorption tendency of five samples on the surface of iron oxide and lime NPs is almost includes a trend. These results show that in the complex process of adsorption, the type of asphaltene (asphaltenes of different origin) dominates the type of metal and lime adsorbents (i.e., there is no effect that changes the tendency of asphaltenes to be adsorbed by iron oxide and lime NPs and we see the same qualitative adsorption trend for all NPs and only the quantitative amount of adsorption differs). Considering the influence of the type of asphaltene on the surface of these NPs, the reasons for the different adsorption amounts of asphaltenes on NPs surface can be attributed to the role of heteroatoms of asphaltenes, the aromaticity of asphaltenes, the size of asphaltene particles and their polarity. According to the results of the elemental analysis of five asphaltenes, it was found that there is a straightforward relation between the nitrogen content and the aromatic nature of asphaltenes with asphaltene adsorption, which can be said that the adsorption of asphaltenes on the surfaces of magnetite NPs, hematite, calcite, and dolomite increases with the increase of these two parameters. Also, we did not find any correlation between the size of the particles and the asphaltenes polarity with their adsorption amount on the surface of magnetite, hematite, calcite, and dolomite NPs. It should be noted that in our recent research, the polarity of asphaltenes was one of the important parameters in the asphaltenes adsorption on quartz NPs^17^. It should be noted that the graph of As-1, As-2, As-4, and As-5 adsorption isotherms on the dolomite NPs surface in Fig. 5 is used from our previous work^6^.

The International Union of Pure and Applied Chemistry (IUPAC)^45^ proposed a classification of adsorption isotherms based on the adsorption of N_2_ at 77 K onto fine solid powders (gas/solid systems), which is shown in Fig. S1. Considering that NPs exist as colloidal aggregates in the continuous phase and depending on the size of the pores of the NPs aggregates and the size and mode of asphaltene aggregates and the nature of their interaction with the NPs, adsorption of asphaltenes onto NPs may follow any of the above types of adsorption isotherms (IUPAC classification). By comparing the isotherms of Fig. 5 with Figure S1 (classification of adsorption isotherms), it was found that the adsorption isotherms of asphaltenes As-1 and As-2 for all NPs (iron oxide and lime NPs) follow type II classification of adsorption isotherms, and the isotherm of the adsorption of asphaltenes As-3, As-4, and As-5 for all NPs follow type IV classification of the adsorption isotherm. Since these categories are usually considered for gas–solid systems, we limited the explanations to this extent. For more information on adsorption isotherm classification (IUPAC), interested readers are referred to the literature studies^24,45^.

**Figure 6:**
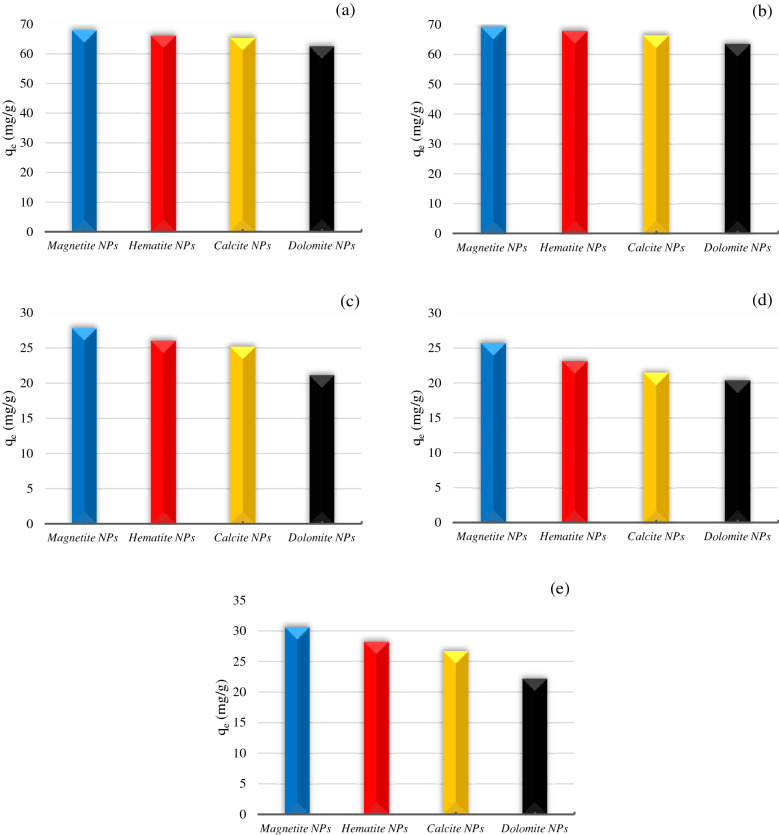
The adsorption amount of each asphaltene (**a**) As-1, (**b**) As-2, (**c**) As-3, (**d**) As-4, (**e**) As-5 onto the magnetite, hematite, calcite, and dolomite NPs at initial concentration of 1000 ppm.

Figure 6 shows the adsorption amount of each asphaltene with magnetite, hematite, calcite, and dolomite NPs. According to Fig. 6, magnetite NPs have the highest adsorption capacity, followed by hematite, calcite, and dolomite (Magnetite NPs > Hematite NPs > Calcite NPs > Dolomite NPs), which shows that iron oxide NPs have more adsorption than lime NPs. Adsorbents (NPs and microparticles) containing iron oxide have a different multi-step adsorption isotherm than other adsorbents (clay and non-clay)^23,46,47^. For this reason, iron oxide NPs had a higher adsorption rate than lime NPs. In addition, adding various iron compounds to asphaltene samples has a considerable impact on their polarity and enhances the asphaltenes tendency to precipitate^48^. The reason for the low adsorption of asphaltenes in lime NPs compared to iron oxide NPs is due to having weak Ca-OH basic groups. Dolomites and calcite considered normally positively charged that could cause weaker interactions between asphaltene functional groups and calcium hydroxyls^23,47^.

### Sensitivity analysis

**Figure 7:**
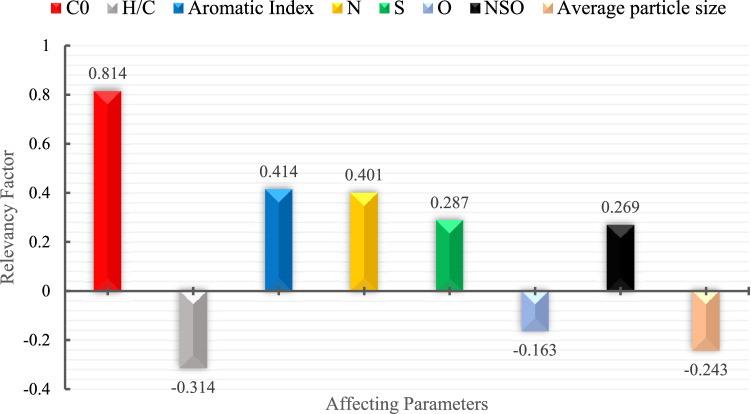
The effect of parameters on the asphaltenes adsorption by magnetite, hematite, calcite, and dolomite NPs.

Considering the wide range of tests and many parameters involved in asphaltene adsorption, using sensitivity analysis is a good option to check the effect of various parameters that may affect the adsorption process. Hence, in order to confirm the parameters affecting the adsorption of asphaltenes on magnetite, hematite, calcite, and dolomite NPs, which was discussed in section "Two-phase asphaltene adsorption experiments", the relevancy factor (r) was used. This special function is used to check the parameters that affect the target response^49,50^. Here, we evaluated the influence of parameters such as asphaltene heteroatoms, asphaltene aromatic nature, average asphaltene particle size, asphaltene polarity, and the initial concentration of asphaltene/toluene solution on the target response (adsorption of asphaltene on magnetite, hematite, calcite, and dolomite NPs) using the relevancy factor. The higher *r* value indicates the greater influence of that parameter on asphaltene adsorption. The relevancy factor is calculated with the following equation^51^:

7 $$r\left({IN}_{i},Q\right)=\frac{\sum_{j=1}^{n}({IN}_{i,j}-{IN}_{m,i})({Q}_{j}-{Q}_{m})}{\sqrt{\sum_{j=1}^{n}{({IN}_{i,j}-{IN}_{m,i})}^{2}\sum_{j=1}^{n}{({Q}_{j}-{Q}_{m})}^{2}}}$$

where *IN*_*i,j*_ and *IN*_*m,i*_ show the *j*th value and mean value of the *i*th input parameter, respectively, where *i* could be heteroatoms, aromatic nature, average asphaltene particle size, polarity, and initial concentration of asphaltene/toluene solution; *Q*_*m*_ denotes the mean value of adsorption of asphaltenes on magnetite, hematite, calcite, and dolomite NPs and *Q*_*j*_ is the *j*th value of asphaltenes adsorption onto the calcite, dolomite, magnetite and hematite NPs. Figure 7 represented the impact of parameters on the adsorption of asphaltenes on the surface of magnetite, hematite, calcite, and dolomite NPs. Considering the shape of the initial concentration of asphaltene/toluene solution, it has the greatest effect on asphaltene adsorption on the surface of magnetite, hematite, calcite, and dolomite NPs, which is quite obvious. The direct relationship between the initial concentration of asphaltene in the solution and its adsorption on different surfaces has been observed in many studies^23,47,52^. Then, the nitrogen content and the aromatic nature of asphaltene, i.e., the aromatic index of asphaltene and the H/C ratio (the value of which is inversely related to the aromatic nature) have the greatest influence on the asphaltenes’ adsorption onto the magnetite, hematite, calcite, and dolomite NPs, which were also mentioned in the previous section and it is also proven in this statistical analysis. Also, the effects of these parameters are consistent with the literature^6,14,26,41,47^. Other parameters (sulfur and asphaltene polarity) have a lesser effect than the mentioned parameters, which has also been observed in the literature^6,26^. The opposite effect on the uptake of asphaltenes by magnetite, hematite, calcite, and dolomite NPs is related to oxygen content, H/C ratio, and average particle size of asphaltenes, which is consistent with the literature^6,26^. Furthermore, the results of the sensitivity analysis are consistent with and confirm the results of section "Two-phase asphaltene adsorption experiments" of this work.

### Three-phase asphaltene adsorption experiments

**Figure 8:**
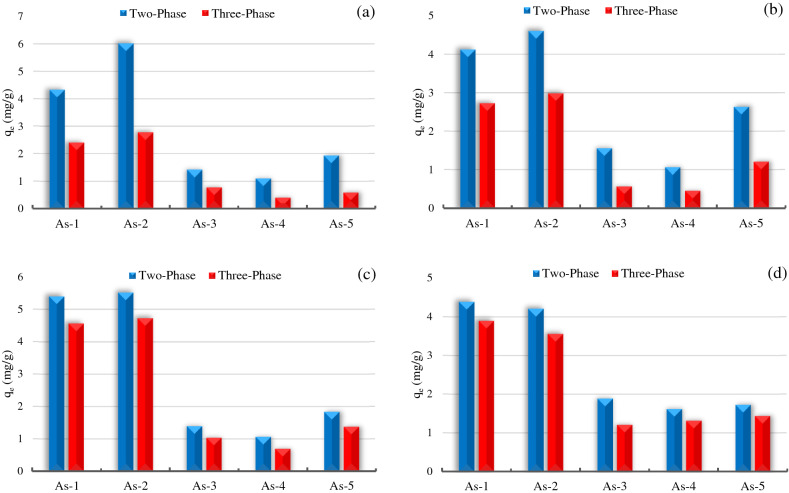
The impact of water on the asphaltenes uptake by (**a**) magnetite NPs, (**b**) hematite NPs, (**c**) calcite NPs, (**d**) dolomite NPs.

Considering that there is water along with oil in the reservoir conditions, as a result, water can have a great influence on asphaltene adsorption. In these experiments, the effect of water on the adsorption of asphaltene on magnetite, hematite, calcite, and dolomite NPs was evaluated. As mentioned earlier, two and three-phase systems contained an equal mass of asphaltenes in the whole solutions (10 ml asphaltene/toluene solution with an initial concentration of 100 ppm for two-phase tests and 5 ml water + 5 ml asphaltene/toluene solution with an initial concentration of 200 ppm for three-phase tests). The results of these tests were presented in Fig. 8. Based on results, the amount of adsorption of five asphaltenes onto the iron oxide and lime NPs in the presence of the aqueous phase (three phases) has decreased significantly compared to the absence of this phase (two phases). This decrease in the amount of asphaltene adsorption in magnetite NPs compared to two-phase is a minimum of 44% associated with As-1 and a maximum of 69% associated with As-5, in hematite NPs, a minimum of 34% associated with As-1 and maximum 58% associated with As-4, in calcite NPs a minimum of 14% is associated with As-2 and a maximum of 34% is associated with As-4, and in dolomite NPs, a minimum of 11% is associated with As-1 and a maximum of 35% is associated with As-3. In the meantime, the reduction of asphaltene adsorption onto the magnetite and hematite NPs (iron oxide NPs) is noticeably higher than that of calcite and dolomite NPs. Hydrogen bonding interactions (H-bonding) are critical for the interactions of asphaltene^…^adsorbent and asphaltene^-^asphaltene, which could be highly altered by the existence of water. It means that as the surfaces of an adsorbent are exposed to moisture, the uptake of sample diminishes as water strongly competes for surface adsorption sites, but does not completely inhibit asphaltene adsorption^23,26^. Water is one of the most powerful liquids for achieving the elimination of polar hydrocarbons adsorbed on iron oxide^26,47,53^. It seems that iron sequestering agents enhance the replacement performance of asphaltene by water, which could be another reason for the lower asphaltenes adsorption onto the iron oxide NPs (magnetite and hematite) in the three-phase system.

### Modeling asphaltene adsorption onto the NPs

Four adsorption isotherm models, namely, Freundlich, Temkin, Langmuir, and Dubinin-Radushkevich were used for modeling the asphaltenes adsorption process on the surface of magnetite, hematite, calcite, and dolomite NPs. Standard deviation (SD) was applied to determine the best adsorption isotherm model. Also, for visual comparison of the models, the responses of the models were drawn graphically (Fig. S2–S5). Table 2 reports the results of the models’ parameters and the SD values calculated from the adsorption data to the response of the models. According to the SD values and the visual comparison of the models, iron oxide NPs i.e., magnetite and hematite in all asphaltenes are consistent with Freundlich and Temkin's model, which shows that the adsorption is physical and multilayered on the surface of these NPs. Also, according to the matching of these models, it shows that the surface of these NPs is heterogeneous. Unlike iron oxide NPs, the models’ responses are different for lime NPs. The results show that the laboratory data of the adsorption of all asphaltenes onto the calcite NPs is consistent with the Freundlich and Temkin models, which, like the adsorption of asphaltenes onto the iron oxide NPs, is physical and multilayered, and the surface of these NPs is also heterogeneous. Regarding the asphaltenes uptake by dolomite NPs, As-1, As-2, and As-4 are consistent with the Freundlich model, which shows physical and multilayer adsorption in the process of adsorption of these asphaltenes onto the dolomite NPs, but As-3 is consistent with the Langmuir model, which shows that the adsorption is in a mono layer and in the form of chemical adsorption. Moreover, As-5 is consistent with the model of Temkin and Dubinin-Radushkevich, according to the parameter E in the Dubinin-Radushkevich model, if E < 8 indicates the nature of physical adsorption, and if 8 < E < 16, it indicates adsorption through ion exchange, and also if E > 16 is, it indicates chemical adsorption, which this parameter for As-5 is smaller than 8, indicating the physical and multilayer adsorption of this asphaltene onto the dolomite NPs. Based on the models, it was determined that physical adsorption renders a significant contribution to the surface adsorption mechanism of asphaltenes on NPs, which is also mentioned in the literature^16^.

**Table 2:** Isotherm modeling results for asphaltenes adsorption by iron oxide and lime NPs.

Model	Parameter	Magnetite NPs					Hematite NPs					Calcite NPs							Dolomite NPs			
		As-1	As-2	As-3	As-4	As-5	As-1	As-2	As-3	As-4	As-5	As-1	As-2	As-3	As-4	As-5	As-1		As-2	As-3	As-4	As-5
Langmuir	q_m_ (mg/g)	28.169	94.3396	19.7628	12.77139	31.34796	26.666	42.19409	29.7619	13.9664	100	52.91005	86.2069	20.9205	18.7969	33.2225	56.4971		45.87156	370.37	36.900	217.39
	K_L_ (L/mg)	0.002281	0.001293	0.000909	0.001013	0.000781	0.002156	0.001944	0.000706	0.000919	0.000328	0.001489	0.001327	0.000824	0.000787	0.000674	0.001433		0.00167	0.0000783	0.000488	0.000139
	SD	0.5286	0.2326	0.4215	0.4594	0.3618	0.5284	0.4618	0.3824	0.4334	0.2484	0.4091	0.3347	0.3853	0.4644	0.3595	0.3937		0.4333	0.1907	0.3082	0.3008
Freundlich	K_F_ (mg/g(L/mg)^1/n^)	0.00517	0.0647	0.002072	0.00088	0.005051	0.004969	0.012688	0.004299	0.001119	0.019788	0.034978	0.043772	0.002235	0.001758	0.005072	0.019611		0.013095	0.016095	0.006636	0.014431
	1/n	1.6274	1.1852	1.4751	1.5868	1.3514	1.608	1.4724	1.3486	1.5328	1.1178	1.2424	1.2542	1.4545	1.4693	1.3371	1.3551		1.4394	1.1216	1.223	1.1592
	SD	0.1410	0.1739	0.1852	0.2120	0.1393	0.1862	0.0703	0.1607	0.2407	0.1820	0.2953	0.0751	0.3264	0.3359	0.3295	0.0658		0.0569	0.2493	0.2201	0.3564
Temkin	b_T_ (J/mol)	35.277	28.417	13.046	12.265	13.826	33.394	33.073	11.81	11.019	11.794	27.178	29.476	12.137	10.444	12.528	28.77		30.781	9.3329	8.893	10.006
	K_T_ (L/mg)	0.0153	0.0228	0.0105	0.0097	0.0112	0.0147	0.0191	0.0112	0.01003	0.0131	0.0185	0.0209	0.0111	0.0112	0.0120	0.0170		0.0164	0.0140	0.0113	0.0147
	SD	0.5206	0.4808	0..3140	0.5592	0.3663	0.6194	0.5269	0.2225	0.4171	0.2968	0.6386	0.4360	0.5271	0.4067	0.6019	0.3453		0.3148	0.3175	0.3469	0.2545
Dubinin-Radushkevich	K_ads_ (mol^2^/J^2^)	0.1781	0.0718	0.4685	0.5289	0.3734	0.1804	0.1714	0.4298	0.5265	0.2554	0.0913	0.0961	0.4663	0.5464	0.3818	0.1761		0.1958	0.3599	0.3654	0.4137
	E (J/mol)	1.6755	2.6388	1.0330	0.9722	1.1571	1.6648	1.7079	1.0785	0.9745	1.3991	2.3401	2.2809	1.0355	0.9565	1.1443	1.6850		1.5980	1.1786	1.1697	1.0993
	q_s_ (mg/g)	36.9844	34.8829	17.0491	14.8321	18.0545	33.7539	42.1315	16.9065	14.0538	16.3822	29.0087	37.2293	16.1916	15.8663	16.8845	39.1968		41.6456	16.3560	12.0011	19.6072
	SD	0.5112	0.4842	0.5366	0.6571	0.5088	0.5601	0.3773	0.4434	0.6488	0.5425	0.6302	0.4370	0.7014	0.5422	0.7230	0.3556		0.3382	0.3902	0.6108	0.2859

In the following, one of the important features of the Langmuir isotherm, the dimensionless separation factor (R_L_), which is calculated by Eq. (8), can be mentioned. This factor is not necessarily derived from the Langmuir equation but it can always be used in it^54^. Although it should be emphasized that R_L_ results are more accurate for adsorption isotherms that correspond to Langmuir, the use of this factor can give us a good view of the adsorption phenomenon. If the value of this parameter is greater than 1, it indicates unfavorable adsorption, equal to 1 linear adsorption, in the range of 1 and 0, favorable adsorption, and equal to 0 indicates irreversible adsorption^26^. Table 3 shows the calculated results of this parameter for all asphaltenes adsorption processes onto the NPs in all the concentrations. The values of this parameter for all modes are in the scope of 0 and 1, which indicates the favorable adsorption of asphaltenes on magnetite, hematite, calcite, and dolomite NPs.

**Table 3:** Results of Langmuir separation factor (R_L_).

Asphaltene	Magnetite NPs				Hematite NPs				Calcite NPs				Dolomite NPs			
	C_0_ 100 ppm	C_0_ 200 ppm	C_0_ 500 ppm	C_0_ 1000 ppm	C_0_ 100 ppm	C_0_ 200 ppm	C_0_ 500 ppm	C_0_ 1000 ppm	C_0_ 100 ppm	C_0_ 200 ppm	C_0_ 500 ppm	C_0_ 1000 ppm	C_0_ 100 ppm	C_0_ 200 ppm	C_0_ 500 ppm	C_0_ 1000 ppm
As-1	0.814	0.686	0.467	0.304	0.822	0.698	0.481	0.316	0.870	0.770	0.573	0.401	0.874	0.777	0.582	0.410
As-2	0.8854	0.794	0.607	0.436	0.837	0.720	0.507	0.339	0.882	0.790	0.601	0.429	0.856	0.749	0.544	0.374
As-3	0.916	0.846	0.687	0.523	0.934	0.876	0.738	0.586	0.923	0.858	0.708	0.548	0.992	0.984	0.962	0.927
As-4	0.908	0.831	0.663	0.496	0.915	0.844	0.685	0.520	0.927	0.863	0.717	0.559	0.953	0.911	0.803	0.671
As-5	0.927	0.864	0.719	0.561	0.968	0.938	0.859	0.753	0.936	0.881	0.748	0.597	0.986	0.973	0.935	0.878

8 $${R}_{L}=\frac{1}{1+{K}_{L}{C}_{0}}$$

## Conclusions

In this work, adsorption of five asphaltene samples with diverse origins on the surface of calcite, dolomite, hematite, and magnetite NPs was investigated. The results showed that iron oxide NPs (magnetite and hematite) and lime NPs (calcite and dolomite) have the same effect on the trend of adsorption of different asphaltenes. Moreover, the most important parameters that affect asphaltene uptake by iron oxide and lime NPs were the asphaltene nitrogen content and the aromaticity of asphaltene, which are directly related to the amount of adsorption; the effect of these two parameters also confirmed by the relevancy factor. Also, the initial concentration of asphaltene/toluene solution showed to be the most important parameter of the asphaltene adsorption onto the NPs. On the other hand, the oxygen content, H/C ratio, and average particle size of asphaltenes had an inverse effect on the adsorption of asphaltenes on NPs, in which the adsorption decreases with the increase in these parameters. In the competition of asphaltene adsorption, iron oxide NPs adsorbed more asphaltene than lime NPs, which are ranked in terms of adsorption capacity as follows: Magnetite NPs > Hematite NPs > Calcite NPs > Dolomite NPs. In the three-phase tests, the amount of asphaltene adsorption in iron oxide and lime NPs decreased, which was higher in iron oxide NPs, the reason for this decrease in adsorption in the three-phase tests is the competition of water for the adsorption of the adsorbent active sites. The results of asphaltene adsorption modeling showed that physical adsorption provides a critical contribution to the surface adsorption mechanism of asphaltenes onto magnetite, hematite, calcite, and dolomite NPs.

## Supplementary Information

Supplementary Information.

## Data Availability

The datasets used during the current study are available within the main manuscript and supplementary file.
