# Supplementary material for: Experimental measurement and modeling of asphaltene adsorption onto iron oxide and lime nanoparticles in the presence and absence of water
Source: Sci Rep. 2023 Jan 4;13:122. doi: 10.1038/s41598-022-27335-z (PMC9813354; doi:10.1038/s41598-022-27335-z)
Supplement: Supplementary file 1 — Supplementary Information. [file 41598_2022_27335_MOESM1_ESM.docx]

**Experimental measurement and modeling of asphaltene adsorption onto iron oxide and lime nanoparticles in the presence and absence of water**

Sajjad Ansari ^1, *^, Mohammad-Reza Mohammadi ^1^, Hamid Bahmaninia ^1^, Abdolhossein Hemmati-Sarapardeh ^1, 2, *^, Mahin Schaffie ^1^, Saeid Norouzi-Apourvari ^1^, Mohammad Ranjbar ^1, 3^

*^1^ Department of Petroleum Engineering, Shahid Bahonar University of Kerman, Kerman, Iran*

*^2^ Key Laboratory of Continental Shale Hydrocarbon Accumulation and Efficient Development, Ministry of Education, Northeast Petroleum University, Daqing 163318, China*

*^3^ Department of Mining Engineering, Shahid Bahonar University of Kerman, Kerman, Iran*

**Supplementary file:**

**Table S1**. FTIR band assignment ^1-3^.

| **Wavenumber, cm-1** | **Functional group** |
| --- | --- |
| 3548, 3430 | A broad and weak band due to O–H (hydroxyl) and N–H (amine) stretching |
| 2923 | Asymmetrical stretching of C-H bond in CH_2_ (methylene) |
| 2852 | Symmetrical stretching of C-H bond in CH_2_ (methylene) |
| 2405 | CO_2_ species in atmosphere |
| 1600 | Stretching of C=C bond in aromatic rings |
| 1460 | Asymmetrical stretching of C=C bond in aromatic rings,  Asymmetrical bending of C-H in CH_3_,  Symmetrical bending of C–H in CH_2_ |
| 1376 | Symmetrical bending of C-H bond in CH_3_ |
| 1315 | Bending of C–H bond in CH_3_ and stretching of C–O bond in carboxylic acid |
| 1030 | Stretching of S=O bond in sulfoxides |
| 911, 864 and 814 | Out of plane bending of C–H bond in aromatic compounds |
| 724 | Out of plane bending of C–H bond in aromatic compounds and bending (rocking type) of C–H in CH_2_ (this pick indicates straight chain alkanes with 4 or more carbon atoms) |


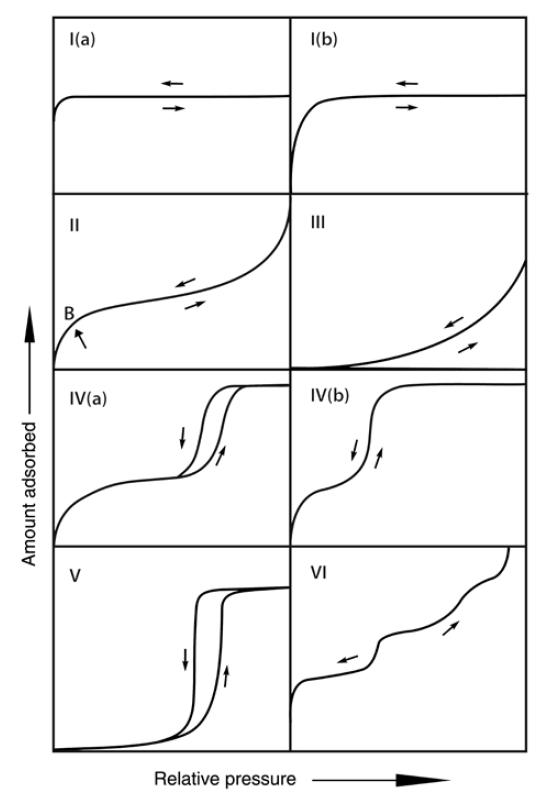


**Figure S1**. IUPAC classification curves for adsorption isotherms ^4,5^.

**Figure S2**. Adsorption isotherm modeling for uptake of all asphaltenes by magnetite NPs.

**Figure S3**. Adsorption isotherm modeling for uptake of all asphaltenes by hematite NPs.

**Figure S4**. Adsorption isotherm modeling for uptake of all asphaltenes by calcite NPs.

**Figure S5**. Adsorption isotherm modeling for uptake of all asphaltenes by dolomite NPs.

**References**

1 Hemmati-Sarapardeh, A., Dabir, B., Ahmadi, M., Mohammadi, A. H. & Husein, M. M. Toward mechanistic understanding of asphaltene aggregation behavior in toluene: the roles of asphaltene structure, aging time, temperature, and ultrasonic radiation. *Journal of Molecular Liquids* **264**, 410-424 (2018).

2 Asemani, M. & Rabbani, A. R. Oil-oil correlation by FTIR spectroscopy of asphaltene samples. *Geosciences Journal* **20**, 273-283 (2016).

3 Pavia, D., Lampman, G., Kriz, G. & Vyvyan, J. (Stanford, CA, USA, 2014).

4 Thommes, M. *et al.* Physisorption of gases, with special reference to the evaluation of surface area and pore size distribution (IUPAC Technical Report). *Pure and applied chemistry* **87**, 1051-1069 (2015).

5 Myers, D. *Surfaces, interfaces, and colloids*. Vol. 415 (Wiley New York, 1999).
